# Effects of Menaquinone-7 on the Bone Health of Growing Rats under Calcium Restriction: New Insights from Microbiome-Metabolomics

**DOI:** 10.3390/nu15153398

**Published:** 2023-07-31

**Authors:** Ya Yuan, Ignatius Man-Yau Szeto, Na Li, Hua Yang, Yunzheng Zhou, Biao Liu, Fang He, Lishi Zhang, Sufang Duan, Jinyao Chen

**Affiliations:** 1 Department of Nutrition and Food Safety, West China School of Public Health and West China Fourth Hospital, Sichuan University, Chengdu 610041, China; scuyaya@163.com (Y.Y.); 13476829658@163.com (N.L.); 15608195382@163.com (Y.Z.); hf18602880124@163.com (F.H.); lishizhang_56@163.com (L.Z.); 2 School of Laboratory Medicine, Chengdu Medical College, Chengdu 610500, China; 3 Food Safety Monitoring and Risk Assessment Key Laboratory of Sichuan Province, Chengdu 610041, China; 4 Yili Maternal and Infant Nutrition Institute (YMINI), Inner Mongolia Yili Industrial Group, Co., Ltd., Beijing 100071, China; szeto@yili.com (I.M.-Y.S.); bliu@yili.com (B.L.); 5 Inner Mongolia Dairy Technology Research Institute Co., Ltd., Hohhot 010110, China; 6 National Center of Technology Innovation for Dairy, Hohhot 013757, China; 7 Department of Nutrition, Renmin Hospital of Wuhan University, Wuhan 430060, China; 8 The Analysis and Assay Center of Sichuan University West China School of Public Health, Sichuan University, Chengdu 610093, China; yangwawa19@163.com

**Keywords:** menaquinone-7, calcium, growing rat, bone mass accumulation, 16s rRNA gene sequencing, metabolomics

## Abstract

Insufficient calcium intake during growth is a global public health concern. The aim of this study was to investigate the effects of dietary menaquinone-7 (MK-7) on bone accrual in growing Sprague–Dawley rats under calcium restriction. Following 13 weeks of treatment, various bone quality parameters, including microarchitecture, were measured. Fecal and cecal samples were subjected to microbiome (16S rRNA gene sequencing) analyses, while metabolomics analysis of the cecum and humerus samples was analyzed based on UHPLC-Q/TOF-MS. We found that calcium deficiency diminished the richness of the microbiome and disrupted microbiome composition, accompanied by an elevation in the relative abundance of *Parasutterella*. Furthermore, calcium insufficiency escalated the level of isovaleric acid and modified the metabolic profiles. MK-7 supplementation significantly increased the cortical thickness, cortical bone area, and the calcium content of the femur. Apart from improving bone calcium deposition and diminishing bone resorption, the mechanisms underlying the beneficial effects of MK on bone quality also involve the modulation of the host’s metabolic pathways and the composition of gut microbiota. The gut–bone axis holds promise as an efficacious target for ameliorating calcium deficiency in children’s bone quality, and MK-7 is a promising dietary supplement from this perspective.

## 1. Introduction

Calcium is a major constituent of bones and plays a crucial role in bone development and maintenance [1]. Inadequate calcium intake during childhood is a globally public health concern [2]. Calcium deficiency among children can lead to result in impaired bone calcification, stunted growth, and elevated risk of various chronic ailments in adulthood, such as osteoporosis. Despite substantial efforts to enhance children’s calcium intake globally, a recent systematic review highlighted the high prevalence of inadequate calcium intake in many countries in South, East, and Southeast Asia [2]. A recent nationwide survey in China reported that the average usual daily calcium intake of children is only one-third of the current recommended intake, and over 96% of children and adolescents aged 4–17 years have insufficient calcium intake [3].

Therefore, in addition to increasing calcium intake, exploring an approach to achieving bone health under circumstances of dietary calcium inadequacy has drawn considerable attention. Furthermore, emerging evidence suggests that micronutrients are involved in the achievement and maintenance of bone integrity [1]. Several studies investigated the effects of the combinations of vitamin K_2_ and D on bone quality [4,5,6,7], achieving quite promising results [4]. However, data are still limited regarding the effects of the combinations of vitamin K_2_ and vitamin D on bone mass accumulation during the growth stage. Additionally, there is a paucity of studies on the impact of this combination in situations where calcium intake is restricted. Menaquinone-7 (MK-7), a vitamin K_2_ homologue, has the highest bioavailability and steady blood levels over time among the homologs [8]. As such, our study’s primary aim was to comprehensively examine the effect of vitamin K_2_ alone and in combination with vitamin D on bone formation in growing Sprague−Dawley (SD) rats fed with a calcium-insufficient diet, which was set according to the actual calcium intake of children in China. Investigations have demonstrated the mechanisms of vitamin K_2_ in maintaining bone health through activating osteocalcin, facilitating calcium deposition, inhibiting osteoclasts, and promoting bone formation. Nonetheless, the precise mechanism has yet to be fully clarified [9,10].

As a result of the considerable attention given to the gut microbiome’s impact on bone health, we became interested in exploring the role of the gut flora in the effect of Menaquinone-7 on bone quality. On the one hand, vitamin K can influence gut microbial community structure and composition [11,12]. On the other hand, the composition of gut microbiota is associated with fecal MK concentrations [11,13]. The literature indicates that microbially synthesized vitamin K contributes to the regulation of bone resorption [14]. Furthermore, research has found that natural products can benefit bone properties by inducing biosynthetic pathways for vitamin K_2_ [15]. Nonetheless, the role of the gut–bone axis in the relationship between MK supplements and bone quality remains unclarified. The consumption of micronutrients can affect the microbiota, as evidenced by alterations in bacterial metabolite profiles, such as short-chain fatty acids (SCFAs) and bile acid derivatives.

In order to investigate the effects of dietary MK-7 on bone accrual and the underlying mechanisms, we simulated a calcium insufficiency model with a 13-week treatment in growing rats, administering MK-7 concurrently. Calcium balance status, microstructural properties, calcium content, and Bone Mineral Density (BMD) were tested to evaluated bone accumulation in the femur. The mechanism of MK-7 in the gut–bone axis was explored by analyzing the microbiome and metabolites of fecal and bone samples. Our study would provide novel insights into the role of MK-7 on bone health in children, but also presents novel avenues for intervention strategies aimed at addressing calcium deficiency in pediatric populations during pivotal growth stages.

## 2. Materials and Methods

### 2.1. Chemicals

Menaquinone-7 (MK) was purchased from Sungen Biotech Co., Ltd. (Santou, Guangzhou, China). Vitamin D_3_ (VD) was purchased from D.BASF (Ballerup, Denmark), 100,000 IU/g. All other chemicals used in this study were of the purest form available commercially, unless otherwise noted.

### 2.2. Animals

Seventy-two male SD rats weighing 55 ± 10 g (3 weeks old, SPF grade) were purchased from Beijing Vital River Laboratory Animal Technology Co., Ltd. (Beijing, China; Certificate No. SCXK [jing] 2016-0006). Animal treatment protocols were conducted with the oversight of the Ethics Committee of West China School of Public Health/West China Fourth Hospital, Sichuan University (Gwll2021067) and were in accordance with the guidance of the Guide for the Care and Use of Laboratory Animals [16]. The rats were kept at the animal laboratory center of West China School of Public Health (Chengdu, Sichuan, China; Certificate No. SYXK [chuan] 2018-011). Rats were housed in cages with wire-mesh bottoms to prevent coprophagy and at 20–23 °C, relative humidity of 60–65%, and a 12/12-h light/dark cycle, with free access to distilled water. Rats were fed a standard AIN-93G diet [17], and were allowed to acclimate to the environment for 2 weeks (day 0). The age of 5 weeks was selected to mimic human bone growth in children.

### 2.3. Treatment

**Figure 1:**
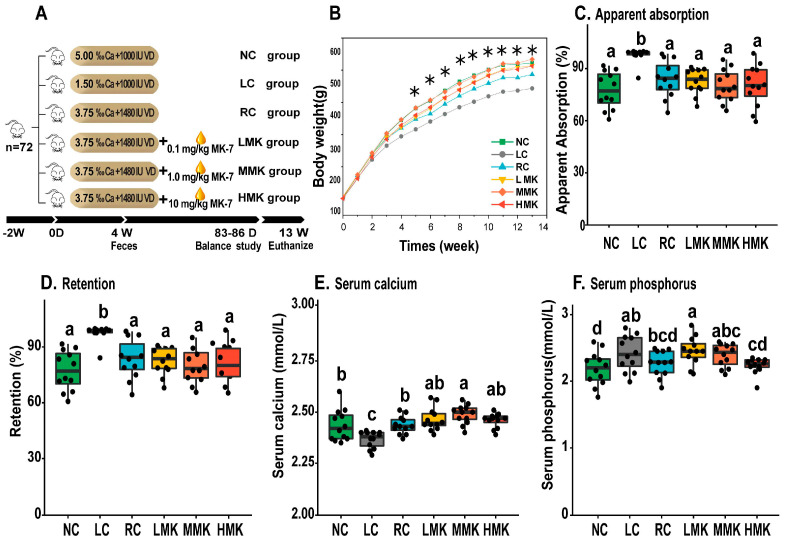
Effects of menaquinone-7 on general condition and calcium balance study of growing rats under calcium restriction (*n* = 12/group). (**A**) Experimental design. (**B**) Variation in body weight over 13 weeks, asterisks (*) signify that the weight of the five groups was significantly higher than the LC group. (**C**,**D**) Apparent absorption and retention of calcium. (**E**,**F**) Serum levels of calcium and phosphorus, as assessed by biochemical methods. Different letters denote statistical differences among groups at *p*  <  0.05, as determined by ANOVA and LSD multiple-comparison test.

After the adaptation period, the rats were randomly assigned to six groups (*n* = 12/group). Figure 1A shows the experimental group. The three control groups were the low-calcium (LC) group, normal-calcium (NC) group and reinforcement control (RC) group. The LC group and NC group, which served as a negative and positive control, were fed with 30% and normal calcium content of the AIN-93G diet, that is, 0.15% and 0.5%, respectively. The RC group and the three MK-7 groups were fed with 75% calcium and 148% VD of the AIN-93G formulation. The three MK-7 groups received daily MK-7 at doses of 0.1, 1.0, and 10.0 mg/kg.bw and were named the low-MK (LMK), medium-MK (MMK), and high-MK (HMK) dose groups, respectively. MK-7 was suspended in corn oil (Yihai Kerry, Shanghai, China) and administered by gavage every day. All treatments were given for 13 weeks.

All diets used in the present study were based on the standard AIN-93G formulation. The only source of dietary calcium was calcium carbonate. All experimental diets were formulated with a calcium-free mineral mix and a VD-free vitamin mix. The dietary content of phosphate and vitamin K_1_ (phylloquinone) in all groups were 3000 mg/kg and 750 µg/kg diet, respectively. Rats had free access to their respective diets.

### 2.4. Biological Sampling

Body weights were measured weekly throughout the study. Fresh feces at day 28 (D28) were collected. The animals were euthanized by an overdose of sodium pentobarbital at the age of 18 weeks. Then, the ceca were quickly separated, and the contents were collected. Blood samples were collected from the abdominal aorta and centrifuged to collect serum samples. Feces, cecum contents, and serum were placed in the cryopreservation tube and stored at −80 °C for analysis. Intact femur and humerus bones were excised, and dissected free of soft tissues. The left femora were dried at 105 °C until a constant weight was obtained. The right femora were fixed in 4% buffered paraformaldehyde for 36 h and soaked in 70% ethanol at 4 °C for imaging analysis. The humeri were wrapped in a tinfoil paper and stored at −80 °C for analysis. Microbiome analyses were conducted on fecal and cecal samples from the NC, LC, RC and HMK groups, while metabolomics analyses were conducted on the cecal contents and humerus samples from the same groups.

### 2.5. Calcium Balance Study

For calcium balance studies, all urine and feces were collected for three consecutive days from all rats separately kept in metabolic cages on D 83–86, respectively, and the feed amount was recorded. The content of calcium in the diet, urine and feces was determined after wet digestion with nitric acid and perchloric acid by flame atomic absorption spectrometry (AAS) (Analytic Jena ZEEnit 700P spectrometer, Analytik Jena, Jena, Germany). The apparent absorption ratio and retention ratio of calcium were calculated using the following equations.

Calcium Apparent Absorption (%) = (calcium intake − fecal calcium excretion)/calcium intake × 100(1)

Calcium Retention (%) = (calcium intake − fecal calcium excretion − urinary calcium excretion)/calcium intake × 100(2)

### 2.6. Determination of Nutrient Levels and Bone Turnover Biomarkers

Serum calcium, phosphorus, and alkaline phosphatase (ALP) were detected by an Olympus AU400 automatic blood biochemical analyzer. The serum levels of uncarboxylated osteocalcin (Glu-OC) and carboxylated osteocalcin (Gla-OC) were measured by commercial dual-antibody ELISA tests (Takara Bio Co., Ltd., Otsu, Japan). The levels of parathyroid hormone (PTH) and insulin-like growth factor (IGF-1) were measured using an ELISA kit purchased from Raybiotech (Peachtree Corners, GA, USA). Serum Type I collagen cross-linked C-terminal telopeptide (CTX) and procollagen type I N-terminal propeptide (PINP) were measured using specific Elabscience (Elabscience, Wuhan, China) ELISA kits. All procedures were conducted according to the manufacturers’ instructions, and absorbance was measured on a microplate reader (Thermo Fisher Multiskan GO, Waltham, MA, USA).

### 2.7. Femoral Calcium Content and BMD Evaluation

After the left femur was dried, the length [18] and the weight of the femur were measured with a Vernier caliper (precision 0.005 mm) and a balance (precision 0.001 g), respectively. After dry ashing at 500 °C in a muffle furnace, the femoral calcium content was determined by AAS as described in the above methods.

The BMD of the right femur was measured in the middle part of the femur with a DXA apparatus (DXS PRO system, Carestream, NY, USA) by using a standard protocol after μCT.

### 2.8. Micro-Computed Tomography (μCT)

The trabecular and cortical architecture of distal femur specimens (*n* = 12 per group) were evaluated using a PerkinElmer μCT (Quantum GX μCT Imaging System, PerkinElmer, Waltham, MA, USA). The μCT analyses were conducted according to current guidelines for the assessment of bone microstructure in rodents by using micro-computed tomography [19]. The femur was scanned with a 72 μm voxel size in all dimensions. The scan conditions were X-ray tube potential of 80 kV and X-ray intensity of 100 μA. Reconstruction was accomplished by PerkinElmer. 3D and 2D analyses were performed using a software CT analyzer. At the same gray value, the region of interest (ROI) was set to a region having the same height of 0.5 mm and a length of 50 slices from the growth plate.

The parameters assessed were total volume (TV, mm^3^), bone volume (BV, mm^3^), bone volume/total volume (BV/TV, %), trabecular thickness (Tb.Th, μm), trabecular separation (Tb.Sp, mm), trabecular number (Tb.N, mm^−1^), cortical thickness (Ct.Th, mm), cortical bone area (Ct.Ar, mm^2^), and cortical bone area fraction (Ct.Ar/Tt.Ar, %), as recommended in the guidelines.

### 2.9. High-Throughput 16S Ribosomal RNA Gene Sequencing and Sequencing Data Analysis

The genomic DNA of feces was extracted using a QIAamp DNA Stool Mini Kit (Tiangen, Beijing, China) according to the manufacturer’s instructions. The integrity and size of the DNA were verified through 1% agarose gel electrophoresis, and DNA concentrations were determined. The 16S rRNA-based amplification process was executed by utilizing the primers 341F (5′-CCTAYGGGRBGCASCAG-3′) and 806R (5′-GGACTACNNGGGTATCTAAT-3′), which are directionally targeting the V3-V4 hypervariable regions of the 16S rRNA gene. The amplicons underwent a purification, quantification, and normalization process before being combined in equal molar concentrations. The sequencing of the pooled amplicons was then conducted using an Illumina Novaseq 6000 sequencing instrument (Illumina, Inc., San Diego, CA, USA).

Effective sequencing data were then clustered into operational taxonomic units (OTUs) with 97% similarity cut-off using USEARCH (version 11). The RDP Classifier (version 2.13) algorithm was used to annotate taxonomic information for each representative sequence using the Silva Database (SSU138). The Mothur program (version 1.30.2) was employed to analyze rarefaction curves and calculate richness estimators and diversity indices. Linear discriminant analysis (LDA) coupled with effect size (LEfSe) was applied to evaluate the differentially abundant taxon.

### 2.10. Cecal SCFA Detection (GC-MS/MS)

The concentrations of acetic acid, butyric acid, caproic acid, isobutyric acid, isovaleric acid, propionic acid, and valeric acid in the ceca were detected by Agilent 7890B-7000D with an internal standard method (Agilent; Santa Clara, CA, USA). Briefly, 20 mg of cecum sample was ground for 10 s with 1 mL of phosphoric acid (0.5% *v*/*v*) and a small steel ball. After being ground three times, the mixture was vortexed for 10 min and then exposed to ultrasonication for 5 min. The supernatant was centrifuged at 12,000 r/min for 10 min. Subsequently, 0.1 mL of the supernatant and 0.5 mL of methyl tert-butyl ether, containing the mix standard solution of SCFAs (CNW Technologies, Shanghai, China), were added to the centrifugal tube. After vortexing and ultrasonication, the mixture was centrifuged at 12,000 r/min for 10 min. After centrifugation, the supernatant was collected, and its components were identified using GC-MS/MS.

### 2.11. Nontargeted Metabolomics Analysis

Nontargeted metabolomics analysis was performed using previously reported methods [20]. Humeri were comminuted after embrittlement in liquid nitrogen. Cecum contents and humerus samples (50 mg) were collected, and 500 µL of −20 °C precooled methanol or 70% ethanol water was added successively. The mixture was agitated for 5 min and centrifuged at 4 °C for 10 min, and the supernatant was removed. Then, 400 μL of 70% methanol water internal standard extractant was added to the sample, and the mixture was shaken at 1500 rpm for 5 min. Afterward, the sample was placed on ice for 15 min. The sample was spun in a centrifuge at 12,000 rpm at 4 °C for 10 min and then 300 μL of the supernatant was collected. The supernatant was placed in a freezer at −20 °C for 30 min. Finally, centrifugation at 12,000 rpm at 4 °C was performed for 3 min, and the supernatant was used for further analysis. The samples were analyzed using an LC/MS Agilent chromatography mass spectrometer (1290 Infinity LC Agilent, CA, USA). The analytical conditions were carried out as described previously [20]. Then, the acquired LC/MS data obtained were converted, deconvoluted, and aligned to match against the freely available MS and Retention Time Index (MSRI) library. For metabolite identification, public databases and self-constructed internal databases were used. Principal component analysis (PCA) and orthogonal partial least squares discriminant analysis (OPLS-DA) was performed in R.

### 2.12. Statistical Analysis

Values are presented as the mean ± standard deviation (mean ± SD). Data analyses were conducted using SPSS 26.0 (SPSS Inc., Chicago, IL, USA) and R version 3.5.1 (R Foundation, Vienna, Austria). Multiple comparisons were checked for normality by the Shapiro–Wilk test before statistical analyses. Data were analyzed by one-way analysis of variance (ANOVA), followed by LSD tests for post-hoc multiple comparisons. Two groups were analyzed using Student’s unpaired t test with Benjamini–Hochberg correction performed. Differential abundances of genera and metabolites were determined by non-parametric tests including the Wilcoxon rank sum test. Bone quality was then tested for association with 16S levels and metabolite intensities using Spearman rank correlation. Differences were considered statistically significant at *p* < 0.05.

## 3. Results

Following 13 weeks of treatment of MK-7 under calcium restriction, various bone quality parameters were examined to assess bone accumulation, and to explore the underlying mechanisms, analyses were performed on the microbiome of cecal contents and feces, along with the metabolomics of both cecal contents and the humerus.

### 3.1. General Condition of Growing Rats

Although partial depilation was observed in several rats in the LC group from the 4-week study to the end of the 13-week study, no abnormal behavior or symptoms were observed in the rats. Figure 1B presents the body weight changes of the six groups. The body weight of the LC group was significantly lower than that of the other five groups from 5 weeks until 13 weeks (*p <* 0.05). The terminal body weight and weight gain of the MK-7 groups were higher than those of the RC group, but the difference was not statistically significant (Table S1). MK-7 administration exerted no significant effect on the coefficients of the heart, liver, and spleen. However, the kidney coefficient in the MMK and HMK groups was lower than that of the RC group but not different from that of the NC group (Table S1), indicating that detrimental effects to the kidneys caused by low calcium intake can be remediated by the combined administration of MK-7 and VD.

### 3.2. Apparent Absorption and Retention of Calcium

The average calcium levels of the NC diet, LC diet and RC diet offered to the rats were 0.49%, 0.14%, and 0.38% diet by analysis, respectively. As expected, the apparent absorption and retention of Ca (%) in the LC group were significantly higher (*p* < 0.05) than those observed in the other five groups. The administration of MK-7 had no significant influence on the absorption and retention of calcium in the balance studies. In addition, the ratios were negatively correlated with the content of calcium in the diet (*p* < 0.05), indicating that calcium intake is the critical factor determining the apparent absorption and retention of calcium. This result is in line with previous studies showing that the absorption and utilization of calcium are negatively correlated with the level of calcium intake [21].

### 3.3. Serum Bone Turnover Parameters

The serum Ca level of the LC group was significantly lower than those observed in the other five groups (*p* < 0.05; Figure 1E). The serum Ca level of the MMK group was significantly higher than that of the RC group (*p* < 0.05; Figure 1E). The serum phosphorus of rats in the RC group was significantly lower than that of the LMK group but had no difference when compared with the that of the rats in the MMK and HMK groups (Figure 1F).

**Figure 2:**
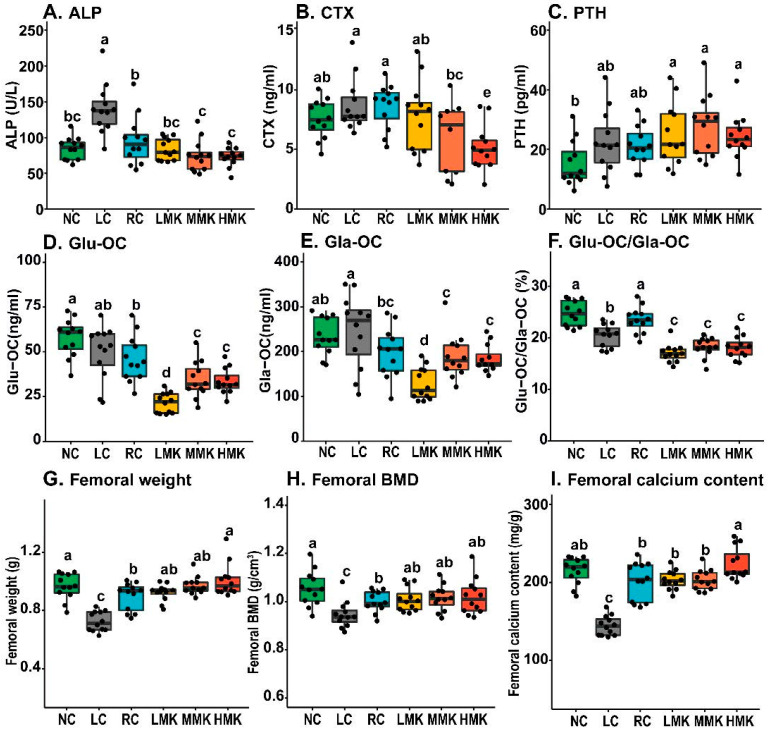
Effects of MK-7 on bone turnover and quality parameters of growing rats under calcium restriction (*n* = 12/group). (**A**) Serum levels of alkaline phosphatase (ALP) were detected by automatic blood biochemical analyzer. (**B**–**F**) Serum levels of Type I collagen cross-linked C-terminal telopeptide (CTX), uncarboxylated osteocalcin (Glu-OC), carboxylated osteocalcin (Gla-OC), and parathyroid hormone (PTH) were measured by ELISA tests. (**G**) Dry femoral weight. (**H**) Femoral BMD was measured by DXA. (**I**) Femoral calcium content was detected by AAS. Different letters denote statistical differences among groups at *p*  <  0.05, as determined by ANOVA and LSD multiple-comparison test.

Serum ALP and CTX levels declined after MK-7 treatment in a dose-effect tendency and decreased significantly in the MMK and HMK groups relative to those in the RC group (Figure 2A,B). The decline in ALP indicated that MK-7 might decrease the stimulation of osteoblast differentiation caused by calcium deficiency by promoting osteoblast maturation [22,23]. The decrease in CTX indicated that MK-7 might decrease osteoclast activity and bone resorption. The serum PTH level of the MK-7 groups had no significant differences when compared with the serum PTH levels of the RC groups (Figure 2C). No significant differences in the levels of PINP and IGF-1 were found among all groups (Table S2). Figure 2D,F show that the serum Glu-OC level and Glu-OC/Gla-OC ratio of the MK-7 groups were significantly lower than those of the RC and LC group (*p* < 0.05), confirming that MK-7 treatment can decrease the Glu-OC/Gla-OC ratio by promoting osteocalcin carboxylation.

### 3.4. Femoral Calcium Content and BMD

Significant decreases in weight, calcium content, and BMD of the femur were exhibited in the LC group (*p* < 0.05; Figure 2G–I). Both the weight and BMD of the femur in the RC group were significantly lower than those in the NC group (*p* < 0.05).

Compared with the RC group, the MK-7 groups showed slight increases in both femoral dry weight and calcium content. The weight and calcium content of HMK were significantly higher than those in the RC group (*p* < 0.05). Even though the MK-7 groups displayed slightly higher BMD than the RC group, the difference was not statistically significant. Overall, MK-7 did not show a positive effect on BMD in growing rats, but 10.0 mg/kg MK-7 increased the femoral dry weight and calcium content.

Notably, no significant differences in BMD were found between the NC and MK-7 groups. However, the values in the RC group were lower than those in the NC group, indicating that the combination of VD and MK-7 compensated for BMD deficits due to dietary calcium inadequacy.

### 3.5. Femoral Microarchitecture Parameters Detected by μCT

**Figure 3:**
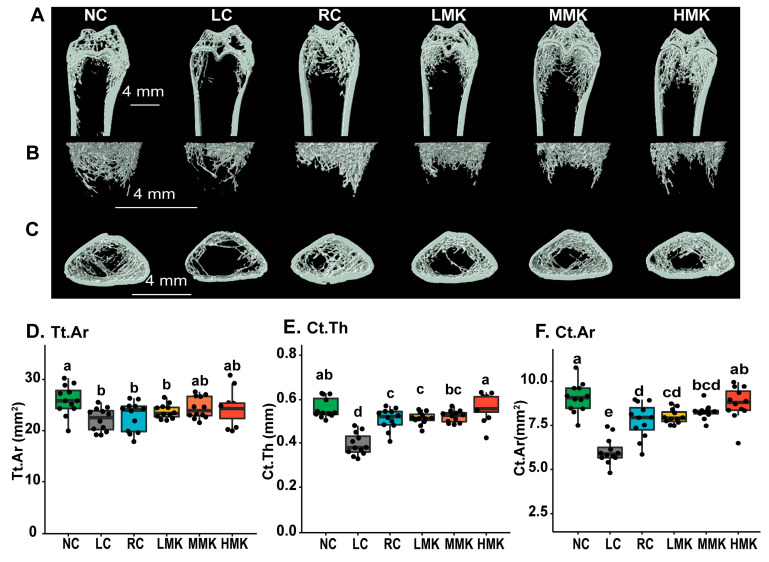
Effects of MK-7 on microarchitecture parameters of growing rats under calcium restriction. (**A**–**C**) Representative three-dimensional images of distal femoral of rats assessed by μCT again. For 3D images, a Bruker Micro-CT Skyscan 1276 system (Kontich, Belgium) was used to image the whole femur with a 6.53 μm voxel size. The scan conditions included an X-ray tube potential of 85 kV and an X-ray intensity of 200 μA. The reconstruction was carried out using Nrecon (version 1.7.4.2). (**D**–**F**) Total cortical bone area (Tt.Ar), cortical thickness (Ct.Th), and cortical bone area (Ct.Ar) of distal femoral was measured by μCT, *n* = 12/group. Different letters denote statistical differences among groups at *p*  <  0.05, as determined by ANOVA and LSD multiple-comparison test.

The μCT data showed differences in the cancellous and cortical bone microarchitecture in the distal femur among the groups (Figure 3A–F, Table 1). Compared with the NC group, LC rats exhibited significant decreases in the number, thickness, and bone volume of trabecular bone (*p* < 0.05). Most observed cancellous microarchitecture parameters were significantly compromised in group with low calcium compared with the normal calcium group (*p* < 0.01). The results showed that calcium deficiency deteriorates the microstructure of the cancellous bone of the distal femur and thereby increases bone fragility and risk of fracture later in life.

The RC group had no difference in the cancellous bone microarchitecture parameters without BV, compared with the NC group. The average cortical thickness (Ct.Th), cortical bone area (Ct.Ar), and total cross-sectional area (Tt.Ar) differed from those in rats in the RC and NC groups (*p* < 0.05). The results are in line with other studies showing that the impact of a calcium-deficient diet during growth is site-specific and more sensitive in the cortical than in the trabecular bone fraction.

Analysis in the distal femur showed that MK-7 intervention slightly increased the TV, BV, BV/TV, and Tb.N, while decreasing the Tb.Th, but the changes were not statistically significant. In the cortical bone, MK-7 intervention increased Tt.Ar and Ct.Ar/Tt.Ar at all doses. In particular, the HMK group significantly increased Ct.Th and Ct.Ar (*p* < 0.05), and no difference was observed when the HMK group was compared with the NC group. This result indicates that a combination of vitamin D and MK-7 can compensate for cortical microarchitecture deficit caused by dietary Ca inadequacy in growing rats.

Additionally, the levels of BV, Ct.Th, Ct.Ar, and Tt.Ar in the RC group were significantly lower than those in the NC group (*p* < 0.05). No differences in these parameters were found between the HMK and RC groups, indicating that the combination of VD and MK-7 can compensate for damage caused by calcium deficiency in the skeletal microarchitecture.

### 3.6. Gut Microbiome Analysis

The gut microbiome was assessed using Illumina NovaSeq sequencing of feces and cecum content derived from three control and HMK rats. The sequencing identified a total of 1606 OTUs across the samples, encompassing 528 species, 258 genera, 106 families, 59 orders, 23 classes, and 16 phyla.

**Figure 4:**
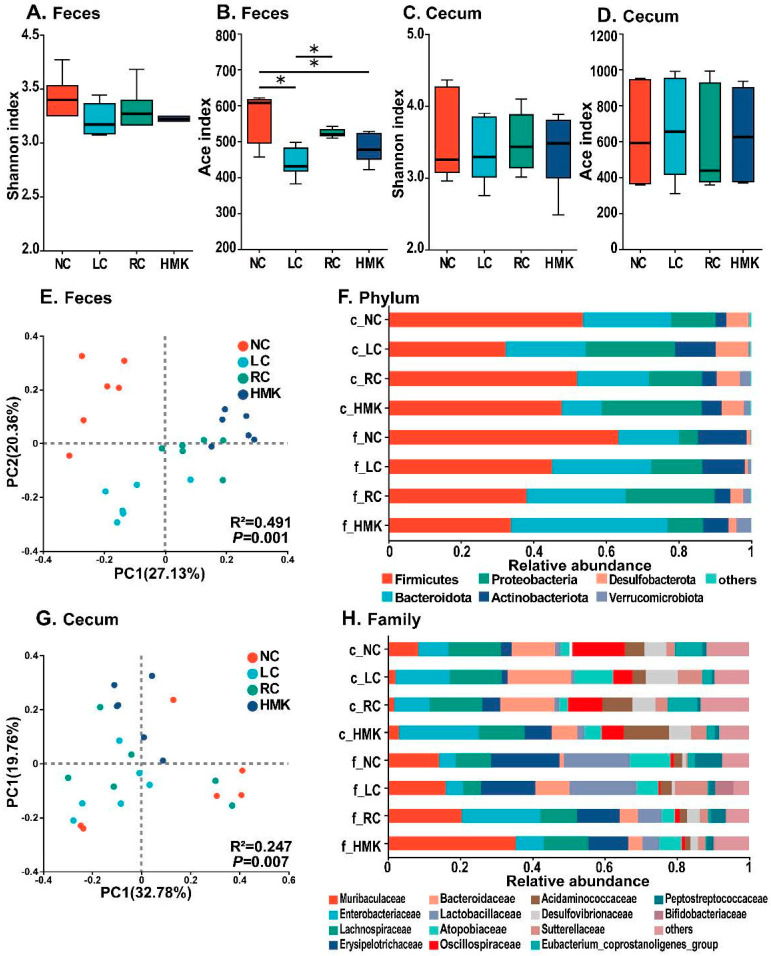
Gut microbiota analysis of growing rats under calcium restriction (*n* = 6/group). (**A**–**D**) Alpha diversity analysis illustrating diversity (Shannon index) and bacterial richness (ACE index) from NC, LC, RC, and HMK groups in both fecal and cecal samples. Asterisks (*) indicate that the index was significantly different between the two groups, as determined by Student’s t test and Benjamini–Hochberg correction. (**E**,**G**) Bray–Curtis Principal Coordinate Analysis (PCoA) plots, representing gut microbiota composition at the operational taxonomic unit level from all four groups in the fecal and cecal samples, respectively. (**F**,**H**) Taxonomic distributions of gut bacterial composition at the phylum and family levels. The prefixes ‘c’ and ‘f’ preceding the bacteria denote that these bacteria were detected in the cecum content and feces.

Alpha diversity analysis of OTUs showed that the Shannon diversity index was consistent across all four groups (Figure 4A,C). However, the ACE diversity index was significantly lower in the LC group compared to the RC and NC group in the fecal sample (*p* < 0.05), while no significant difference was found in the cecal sample (Figure 4B,D). Notably, the alpha diversity indices did not exhibit any significant disparity between the RC and HMK groups. At the phylum level (Figure 4F), *Firmicutes*, *Bacteroidetes*, and *Proteobacteria* were the main predominant phyla, accounting for over 80% of the gut microbiota. At the family level (Figure 4H), the fecal and cecal microbiota were predominantly composed of *Enterobacteriaceae*, *Muribaculaceae*, *Lachnospiraceae*, *Erysipelotrichaceae*, and *Bacteroidaceae.* These accounted for more than 58.56% of the gut microbiota.

The PCoA diagram based on Bray–Curtis distance demonstrated that repeat samples within each group clustered together (Figure 4E,G). The LC groups was categorized into distinct clusters compared to the other three groups, indicating that the restriction of calcium influenced the microbial communities. Further PERMANOVA analysis confirmed that fecal microbial communities significantly varied among the four groups (R^2^ = 0.491, *p* < 0.01), as did the cecal microbial communities (R^2^ = 0.247, *p* < 0.01).

**Figure 5:**
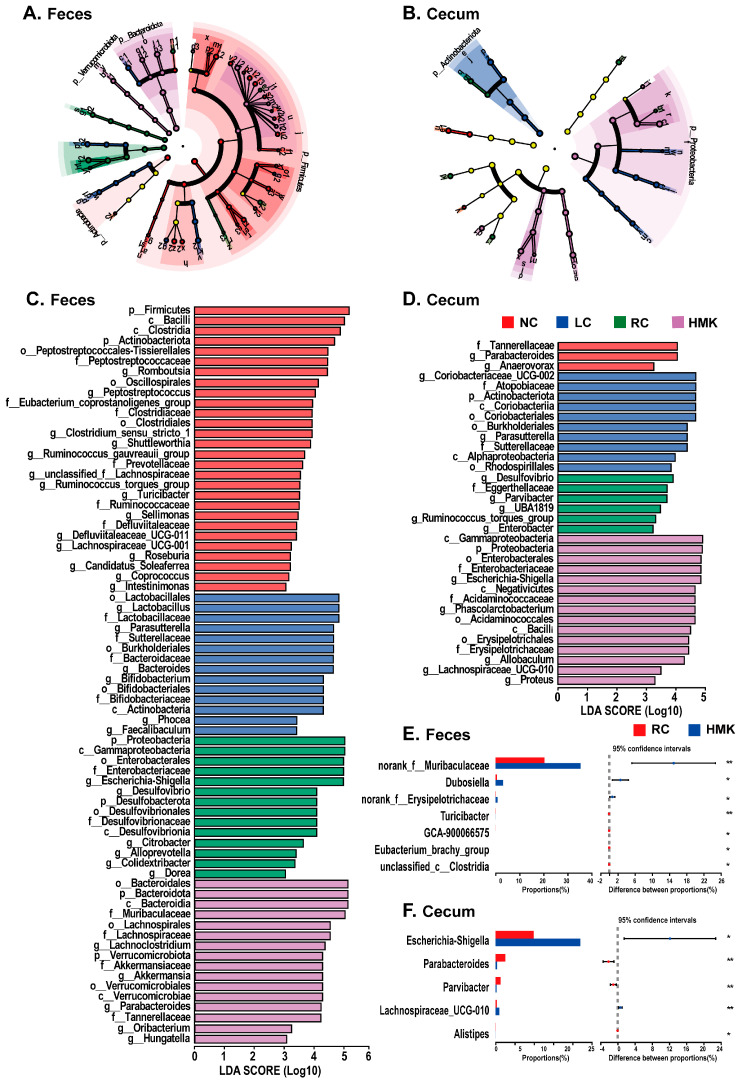
Identification of the characteristic bacterial taxa following MK-7 supplementation in calcium-deficient growing rats (*n* = 6/group). (**A**,**B**) Taxonomic cladograms generated from phylum-to-genus LEfSe analysis for the NC, LC, RC, and HMK groups in the fecal and cecal samples, respectively. (**C**,**D**) Differentially represented bacterial taxonomies identified by LEfSe analysis with an LDA score >3 among all four groups in the fecal and cecal samples, respectively. (**E**,**F**) Analysis of differences bacterial at the genus level between the RC and HMK group in the fecal and cecal samples (*t*-test), respectively. Asterisks (*) indicate that the bacteria was significantly different between the RC and HMK group, as determined by Student’s *t* test and Benjamini–Hochberg correction, with a *p* value of less than 0.05 (*) and 0.01 (**).

As shown in the LEfSe analysis and the cladogram (Figure 5A,B), the specific phylo-types that were responsive to calcium deficiency were *o_Burkholderiales*, *f*_*Sutterellaceae*, and *g__Parasutterella* in both feces and cecum contents. Notably, the RC group was characterized by *g_ Desulfovibrio*. Meanwhile, the specific phylotypes that were responsive to MK-7 supplementation included *Ruminococcus torques group*, *Dubosiella*, *norank_f_Erysipelotrichaceae*, and *norank_f_Muribaculaceae* (from the family *Muribaculaceae*) in the feces. Furthermore, *Parvibacter*, *Parabacteroides* (from the family *Tannerellaceae*), and *Escherichia-Shigella* were identified as biomarkers responsive to MK-7 supplementation in the cecum microbiome (Figure 5C,D). The RC group was characterized by *Rothia* (from the class *Actinobacteria* and family *Micrococcaceae*), *Escherichia-Shigella*, and *Morganella* (from the class *Gammaproteobacteria* and family *Enterobacteriaceae*) in the cecum.

Rats administered with MK-7 had a higher relative abundance of *Dubosiella*, *norank_f_Erysipelotrichaceae*, and *norank_f_Muribaculaceae* and lower abundance of *GCA-900066575* than those of the RC group in the feces (*p* < 0.05) (Figure 5E,F). Rats administered with MK-7 had a higher relative abundance of *Escherichia-Shigella* and *Lachnospiraceae_UCG-010* and lower abundance of *Parvibacter*, *Parabacteroides*, and *Alistipes* than those of the RC group in the cecum contents (*p* < 0.05).

### 3.7. Metabolic Profile of Cecum Content and Humerus

**Figure 6:**
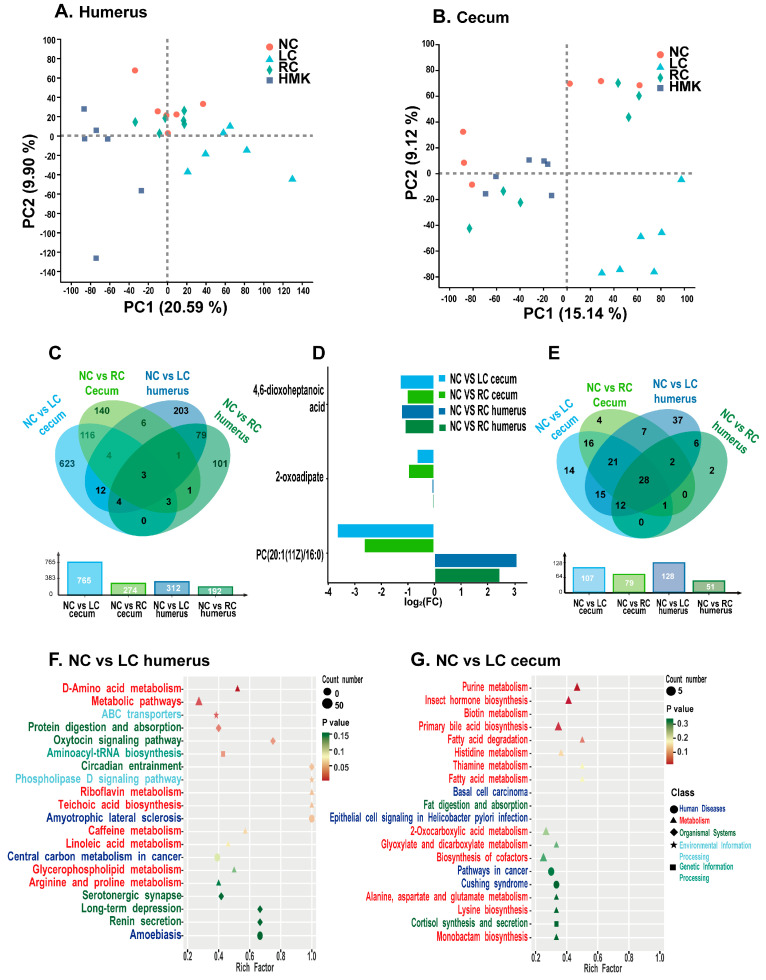
Metabolic profile of growing rats under calcium restriction (*n* = 6/group). (**A**,**B**) PCA plot from NC, LC, RC, and HMK groups in both humerus and cecum content samples. (**C**) Venn diagram summarizing significantly overlapping changed metabolites across two sample types between the NC and LC or RC group. The significantly changed metabolites were screened out according to the value of variable importance in projection (VIP) and *p* value (VIP ≥ 1, *p* < 0.05). (**D**) Bar plots showing three significantly overlapping altered metabolites, indicated by log2 fold change. (**E**) Venn diagram summarizing overlapped metabolic pathways across two samples. (**F**,**G**) Enrichment bubble plot showing the top 20 enriched KEGG pathways ranked by *p* value from NC and LC groups using humeral and cecal samples. The color and shape of the bubble indicate the *p* value and the classification of the enriched KEGG pathway.

In the cecum contents of the RC and HMK rats, a total of 24,523 reproducible peaks, or features, were detected. Among these peaks, 3591 corresponded to previously identified metabolites. In the humerus, 14,880 peaks were detected, of which 1342 were previously identified metabolites. As PCA showed, the metabolomics profiles of the cecum content and humerus reveal obvious separations between the LC group and the other three groups (Figure 6A,B). Moreover, an obvious separation trend among the four groups is visible in the OPLS-DA score plots of different tissues, illustrating evident differences in their cecum and humerus metabolite profiles (Figure S1A–D). Significant collections of metabolites were obtained by applying the criteria of VIP ≥ 1 and *p* < 0.05.

Analysis was conducted to examine the metabolic profile differences between the NC group and either the LC or RC group. Relative to the NC group, the LC group exhibited 765 and 312 distinct metabolites in their cecum content and humerus, respectively (Figure 6C). Similarly, compared to the NC group, the RC group exhibited 274 and 192 differential metabolites in the two respective samples. The Venn diagrams illustrate that three metabolites, specifically 4,6-dioxoheptanoic acid, 2-oxoadipate, and PC(20:1(11Z)/16:0), overlapped between the NC and either LC or RC groups in both samples (Figure 6C). Compared to the NC group, both LC and RC groups exhibited a marked reduction in the levels of 4,6-dioxoheptanoic acid and 2-oxoadipic acid across both samples. Notably, a marked reduction in the concentration of PC(20:1(11Z)/16:0) was observed in the cecum content of both the LC and RC groups, in contrast with the NC group. Conversely, a significant elevation in the PC(20:1(11Z)/16:0) level was detected in the humerus. The fold changes of these three overlapping metabolites are depicted in the bar diagram (Figure 6D).

The Venn diagrams illustrate that 28 of the metabolic pathways exhibited overlapped between the NC and either LC or RC group across both samples (Figure 6E). Notably, nine of these pathways were associated with amino acid metabolism. The metabolic pathways with *p* value < 0.05 and an impact factor > 0.1 were considered the most relevant pathways (Figure 6F,G). Important metabolic pathways in the humerus, observed between the NC and LC group, included D-Amino acid metabolism, metabolic pathways, ABC transporters, protein digestion and absorption, oxytocin signaling pathway, and aminoacyl-tRNA biosynthesis. Additionally, purine metabolism, insect hormone biosynthesis, and biotin metabolism were distinguished as important metabolic pathways in the cecum content. The important metabolic pathway in the humerus between the NC and RC group was caffeine metabolism.

**Figure 7:**
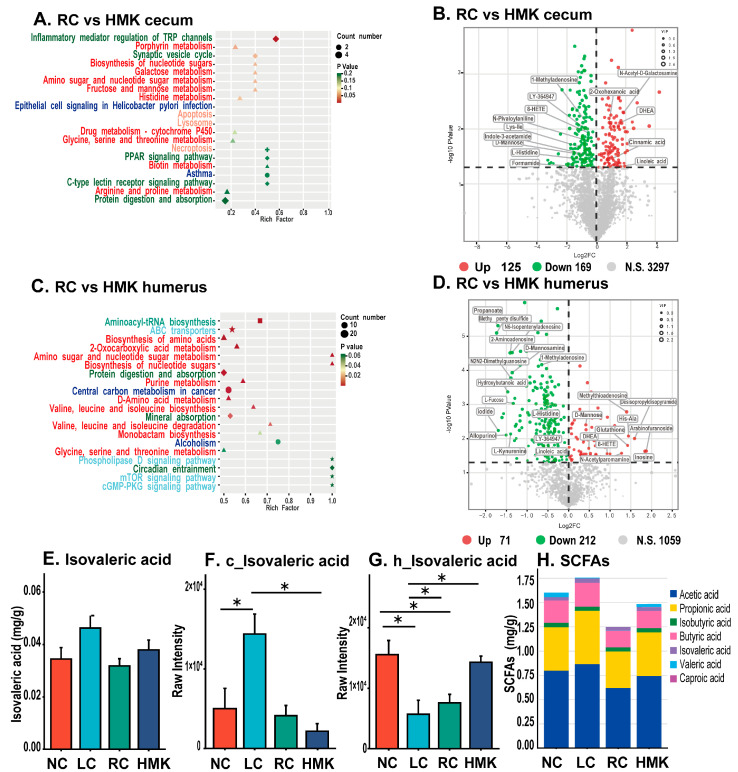
Metabolic profile of growing rats under MK-7 supplementation (*n* = 6/group). (**A**,**C**) Enrichment bubble plot showing the top 20 enriched KEGG pathways ranked by *p* value from RC and HMK groups in the humeral and cecum content samples, respectively. The color and shape of the bubble indicate the *p* value and the classification of the enriched KEGG pathway. (**B**,**D**) Volcano plots presenting the differences between the RC and HMK groups’ cecum and humerus samples. The significantly increased (red) or decreased (green) metabolites were screened out according to VIP ≥ 1 and *p* < 0.05. (**E**,**H**) Levels of cecal isovaleric acid (IVA) and SCFAs were assessed via GC-MS/MS (ANOVA). (**F**,**G**) Raw intensity of IVA in the cecum content and humeral samples were evaluated (*t*-test), respectively. The prefixes ‘c’ and ‘h’ preceding the metabolites denote that these metabolites were detected in the cecum content and humerus, respectively. Asterisks (*) indicate that the metabolite was significantly different between the two groups, as determined by Student’s *t* test and Benjamini–Hochberg correction.

Further analysis was conducted on the metabolic profile of the RC and HMK groups to enhance comprehension of the mechanism by which MK-7 affects bone quality. The cecum content collected from the HMK group exhibited a decrease in 169 metabolites, while an increase in 125 metabolites was observed when compared to the RC group (Figure 7B). Likewise, in the humerus, reductions in 212 metabolites and an increase in 71 metabolites were noted (Figure 7D). Furthermore, the KEGG pathway enrichment results showed that most of the statistically significant metabolic alterations belonged to amino acid and lipid metabolism.

Important metabolic pathways in the humerus, discerned between the RC and HMK groups, included aminoacyl-tRNA biosynthesis, ABC transporters, biosynthesis of amino acids, 2-oxocarboxylic acid metabolism, amino sugar and nucleotide sugar metabolism, biosynthesis of nucleotide sugars, protein digestion and absorption, purine metabolism, D-Amino acid metabolism, central carbon metabolism in cancer, valine leucine and isoleucine biosynthesis, mineral absorption, valine leucine and isoleucine degradation, and monobactam biosynthesis (Figure 7C). Notably, inflammatory mediator regulation of TRP channels emerged as a significant metabolic pathway in the cecum content (Figure 7A).

### 3.8. Cecal SCFA Profile

The concentration of isovaleric acid (IVA) in the LC group was observed to be the highest, and higher than that of the NC, RC and HMK groups (Figure 7E). This finding aligns with the results of the metabolomic analysis (Figure 7F,G). No significant differences were observed in the concentrations of the other six types of SCFAs in the cecum content among all groups (Figure 7H).

### 3.9. Correlation Analysis

**Figure 8:**
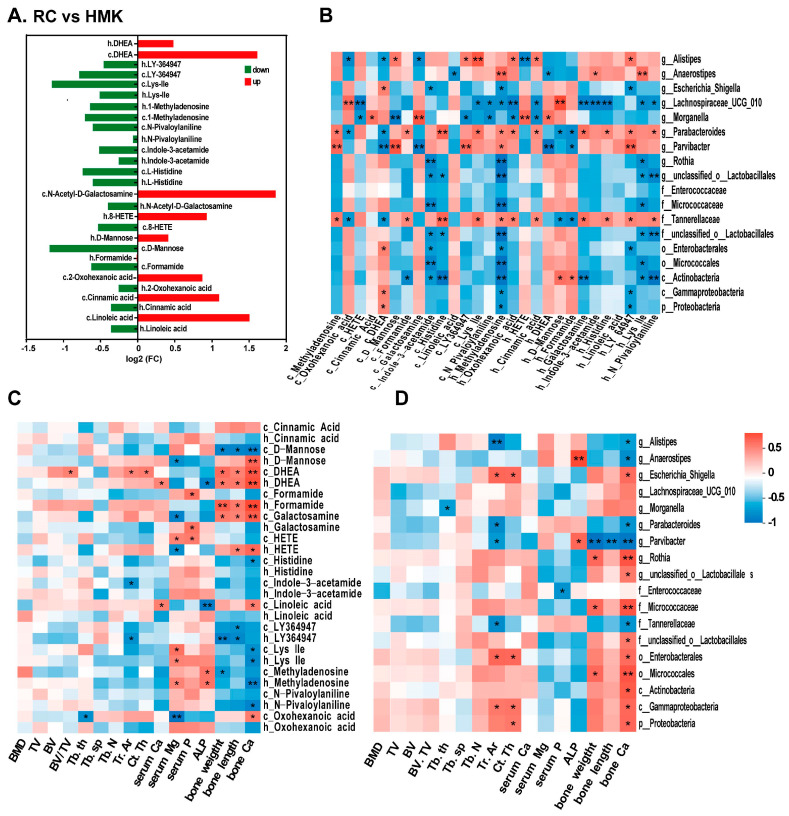
Correlation analysis of the gut microbiota, metabolites, and bone quality of growing rats under calcium restriction (*n* = 6). (**A**) Effects of MK-7 on the overlapped significantly changed metabolites in the cecum and humerus samples. (**B**) Correlation analysis of overlapped significantly changed metabolites and cecum microbiota biomarkers treated with MK-7. (**C**) Correlation analysis of overlapped significant changes metabolites and bone quality treated with MK-7. (**D**) Correlation analysis of bone quality and cecum microbiota biomarker treated by MK-7. Different color blocks represent Spearman correlation coefficients, red indicates positive correlation, and blue indicates negative correlation. * *p* < 0.05; ** *p* < 0.01. The prefixes ‘c’ and ‘h’ preceding the metabolites denote that these metabolites were detected in the cecum content and feces.

To investigate the potential mechanism of MK-7, Spearman’s correlation analysis was conducted for the RC and HMK groups. Among the cecum and humerus samples, 49 metabolites overlapped, with 14 exhibiting significant changes (Figure S1G). The fold changes of these 14 overlapping metabolites are displayed in the bar diagram (Figure 8A). When comparing HMK rats to RC rats, there was a decrease in the levels of 4-(3-pyridin-2-yl-1H-pyrazol-4-yl)quinoline (LY-364947), Lys-Ile, 1-methyladenosine, N-pivaloylaniline, indole-3-acetamide, and L-histidine, whereas the levels of 5-androsten-3β-ol-17-one (DHEA) significantly increased in both humerus and cecum content. Additionally, DHEA was one of the two overlapping metabolites between the HMK and either the LC or NC group in the two samples.

Correlation analyses were performed on bone parameters, the 14 overlapped metabolites, and differentially abundant bacterial genera (based on LEfSe analysis) in the HMK and RC groups using the Spearman correlation coefficient test (Figure 8B–D, Figure S2A,B). The relative abundance of *Proteobacteria*, *Gammaproteobacteria*, *Tannerellaceae*, and *Parvibacter*, and other flora present in the cecum, exhibited a correlation with the femur’s calcium content (*p* < 0.05). The relative abundance of *GCA-900066575* in the feces showed a correlation with the femoral calcium content (*p* < 0.05), and femoral calcium content positively correlated with several significantly changed metabolites.

Interestingly, the level of DHEA positively correlated with calcium content, length, and dry weight of the femur (R > 0.6, *p* < 0.05), suggesting a strong association between DHEA and MK-7 supplementation. An examination was conducted of the correlation between DHEA and the biomarkers of cecum and fecal microbiota. The relative abundance of *Parvibacter* and *GCA-900066575* negatively correlated with DHEA levels in the cecum and humerus, suggesting that the gut microbiota may further influence bone formation by regulating sex hormones (*p* < 0.05).

## 4. Discussion

Calcium-deficient rat models were successfully established by providing low-calcium diets (0.15% or 0.375%) to growing male SD rats for a duration of 13 weeks. A severe lack of calcium (0.15%) during growth was detrimental to the quality and microarchitecture of bones and general condition. These results were consistent with previous studies [24] and highlight once again the importance of adequate calcium intake for maximizing peak bone mass during growth [24]. Although vitamin D_3_ supplementation could partially compensate for the deleterious effects of moderate calcium deficiency (0.375% in diet) on bone by increasing absorption, BMD and cortical architecture was not fully recovered. These finding are in line with previous studies indicating that high serum 1alpha-25(OH)VD levels do not compensate for insufficient calcium intake [25].

Calcium supplementation has been shown to modulate gut microbiota in a prebiotic manner [26] and increase community diversity [27]. However, the effects of dietary calcium on gut microbiota remain largely unexplored [28]. Our research demonstrated that calcium insufficiency can reduce fecal bacterial richness and disrupt their composition, which aligns with previous findings. The significant low-calcium-related increase in the relative abundance of genus *Parasutterella* in the feces and cecum content is noteworthy, as this genus plays an active role in bile acid metabolism and homeostasis [29]. *Parasutterella* has been observed to cause notable changes in microbial-derived metabolites, including amino acid, purine, and bile acid derivatives [29]. Interestingly, the levels of cholic acid, a type of bile acid, in the LC group were higher than those in the NC and other groups, in both the cecum contents and humerus, according to the metabolomics analysis (Figure S1J,K). Our research demonstrated that insufficient calcium intake could lead to abnormal metabolism of amino acid and purine, which is consistent with previous research [30,31,32]. Furthermore, the level of IVA in the LC group was higher than that in the NC, RC, and HMK groups in the cecum content, according to metabolomics analysis. Concordantly, the IVA concentration of the LC group was higher than that of the NC, RC, and HMK group. SCFAs have previously been identified as key mediators in the gut-bone signaling axis. IVA has been reported to suppress osteoclast differentiation both in vitro and in ovariectomized (OVX) models [33]. Interestingly, although the LC group exhibited the highest levels of IVA in the cecum content sample, the corresponding humeral sample from the same group displayed the lowest levels of IVA. The humerus of the NC group showed a higher concentration of IVA compared to the value in the LC group, indicating more potent inhibition of osteoclast differentiation. This observation aligns with the observed CTX levels. However, the divergent response of IVA in bone and intestinal content warrants further investigation. Collectively, our results demonstrate that calcium deficiency can disrupt the gut microbiome and metabolic profile, leading to alterations in body metabolism.

Thirteen-week daily administration of 10.0 mg/kg.bw MK-7 increased the femoral calcium content and intensified cortical architecture in growing male rats. This morphological intensification of the cortical bone, consistent with the increase in calcium content, showed that MK-7 benefits cortical bone in growing subjects [25]. The levels of Gla-OC and Glu-OC increased within a dose range of 0.1–1.0 mg/kg MK-7. Hence, the lack of difference in the Glu-OC/Gla-OC ratio between the LMK and MMK groups is reasonable. The same declining tendency in the levels of serum Gla-OC and Glu-OC implied that they are not the proper indicators of vitamin K status, as indicated in other reviews [34,35]. We speculated that the response of the Glu-OC/Gla-OC ratio to MK-7 intake may be nonlinear, with a plateauing decrease above 0.1 mg/kg.bw. This finding confirmed that MK-7 can stimulate the secretion of osteocalcin, a gamma-carboxylated protein that can effectively bind calcium deposits in the bone matrix [36,37]. In addition, we found that MK-7 administration decreased the levels of ALP and CTX. This result suggested that MK-7 could stimulate bone formation and suppress bone resorption, as previous in vivo and in vitro studies have proposed [22]. However, the promotion of bone accumulation at an effective dose of 10.0 mg/kg.bw in our study was considerably more extensive than that reported in other studies [7,22]. These studies have suggested that MK-7 can activate OC at doses in the microgram range [7,22,37], but the impacts of MK-7 on bone at that dose remain inconsistent. Doses in the microgram range of MK-7 or 22 mg/kg of MK-4 [18] may not be sufficiently effective during growth as children exhibit much higher bone metabolism than adults [38]. Our results indicate that a high dosage may be necessary when bone metabolism accelerates.

The beneficial impact of vitamin K on bones is acknowledged, yet its molecular mechanisms are not fully comprehended [35,39,40]. Given that the Glu-OC/Gla-OC ratio did not decline as MK-7 intake increased, we speculated that the effects of high doses of MK-7 may be mediated by mechanisms other than increasing osteocalcin carboxylation and bone turnover [38]. The similar trend observed in the levels of Gla-OC, and Glu-OC in response to MK-7 supports this hypothesis. The findings also insinuate that vitamin D and MK-7 may reciprocally influence each other through yet unidentified mechanisms, necessitating further exploration. Moreover, the beneficial effects on the microstructure parameters of cortical bone indicate that MK-7 exerts its effects through other mechanisms.

The cancellous and cortical parameters and BMD in the RC group were disrupted and decreased compared with those in the NC group, whereas no difference was found between the HMK and NC groups. That is, the gaps of BMD caused by calcium-insufficient intake to the deteriorated bone accrual were partly bridged by the long-term combined administration of MK-7 and VD in young male young SD rats. These implications are in agreement with previous observational clinical studies [41], which showed that vitamin K_2_ (MK-4), especially when combined with 1,25-(OH)_2_D_3_, can activate bone formation in female premenopausal patients. Vitamin D can enhance vitamin K-dependent bone protein levels [42] and activate bone formation in vitro [43]. The underlying mechanism of bone mineralization induced by vitamin K in the presence of 1,25(OH)D is different from that induced by vitamin K alone [44]. As products of the combination of VD and vitamin K_2_ have promising effects, our findings offer valuable in vivo information and might provide a novel method of effective calcium utilization in children with low calcium intake.

No significant differences in flora diversity and SCFAs concentration in the rat cecum between the RC and HMK groups were observed. Nevertheless, the significantly changed PCoA profile and notable variation in family-level composition showed that MK-7 significantly modulates the gut microbial communities. A recent study found no change in cecal microbiota occurred after MK-4 or MK-9 supplementation in male mice [11]. Collectively, these results indicate that MK-7 influences the composition of the colon microbiota, which is dominated by obligate anaerobic bacteria. We found that 10 mg/kg.bw MK-7 decreased the abundance of *Escherichia coli* and increased the abundance of *Bacteroides fragilis* in the feces. *Escherichia coli* and *Bacteroides fragilis* can manufacture MKs in the large intestine under anaerobic conditions [45,46]. Supplementation with MK-4 or MK-9 reduced the relative abundance of cecal *Bacteroides* and *Ruminococcus_1* while increasing that of *Lactobacillus* in female mice compared with the VK-deficient group [11]. Furthermore, the abundance of *Escherichia-Shigella* in the feces was much higher in the RC group than in the LC and NC groups, which may be related to the level of vitamin D. Overall, our finding suggested that MK influences gut microbiota composition and the abundance of microbes that synthesize MKn. Further studies are needed to explain the mechanism by which MK affects the gut microbiome.

Sex-specific differences have been observed in the gut microbiota and remodeling response to dietary vitamin K in mice [47]. MK supplementation exerted significant effects that reduce bone loss in postmenopausal women, but its effects in men are unclear [48]. Our metabolic profile analysis showed that the levels of DHEA significantly increased in the humeri and ceca of rats supplemented with MK-7 and were positively correlated with bone quality. DHEA increases BMD in OVX rat models and older females [49]. However, DHEA supplementation has demonstrated less consistent results in men. Furthermore, the gut microbiota can affect sex hormones, such as androgen. DHEA is a precursor for androgen and estrogen. Probiotics can increase testosterone levels in aging mice, and transplanting the gut microbiota of male mice to juvenile female mice significantly increases serum testosterone level [50]. Our study showed that the relative abundance of *Parvibacter* and *GCA-900066575* was inversely proportional to the levels of humeral and cecal DHEA and femoral calcium content. *GCA-900066575* is positively correlated with gonadotropin-releasing hormone and may thus promote puberty [51], suggesting that increase in DHEA and the alteration of the gut microbiome may be the reasons that MK-7 is less effective in males.

Significantly, the pathway of protein digestion and absorption, as well as aminoacyl-tRNA biosynthesis, exhibited downregulation in the presence of calcium deficiency (Table S3). Conversely, the supplementation of MK-7 notably upregulated these pathways. It has been documented that the aminoacyl-tRNA biosynthesis pathway is dysregulated in patients with Low Bone Mineral Density and OVX rats [52,53,54]. Additionally, purine metabolism disruptions were observed in response to calcium deficiency. Interestingly, MK-7 supplementation exhibited a considerable downregulating effect on purine metabolism (Table S2). Prior studies have linked purine metabolism to calcium deficiency [31,32] and emphasized its important role in osteoporosis development [55]. Disturbances in purine metabolism can lead to reduced osteoclastogenesis and intrinsic osteoblast function defects, subsequently resulting in low bone formation [56]. In our study, MK-7 increased the levels of inosine, hypoxanthine, guanosine, and adenosine while decreasing the uric acid level—a condition otherwise heightened by calcium deficiency. These metabolites partake in the salvage pathway of purine nucleotides. Thus, we cautiously propose that MK-7′s role in regulating calcium deposition may be associated with its ability to boost the purine metabolism’s salvage pathway.

## 5. Conclusions

The establishment of a calcium-deficient rat model was attained through the implementation of a low-calcium diet (0.15%) in growing male Sprague–Dawley rats for a duration of 13 weeks. Calcium deficiency diminished the diversity of fecal bacteria and disrupted their composition, accompanied by an elevation in the relative abundance of *Parasutterella*. Furthermore, calcium insufficiency escalated the level of isovaleric acid and modified the metabolic profiles of the cecum content and humerus, primarily affecting the metabolic pathway governed by amino acid metabolism. We found that the adverse femoral outcomes in cortical bone and BMD caused by a 25% inadequacy of calcium were partially attenuated by the combined administration of VD and MK-7, and 10.0 mg/kg.bw of MK-7 can significantly intensify cortical accrual and increase calcium content in femurs in this rat model, and the gut microbiota was enhanced as well. Our findings provide insightful evidence of the bone-enhancing effects of MK-7 in growing subjects, highlighting the necessity of future studies on the effects of the combinations of vitamin D and K on bone health in growing subjects. The results indicate that the gut–bone axis holds promise as an efficacious target for ameliorating calcium deficiency in children’s bone quality. In conclusion, the modification in the gut microbiota and humeral metabolism arising from disparate calcium and MK-7 intake levels could furnish invaluable insight into the gut-bone axis.

## Figures and Tables

**Table 1:** Effect of MK-7 on femoral microarchitecture parameters of growing rats under calcium restriction.

Group	NC	LC	RC	LMK	MMK	HMK
TV (m^3^)	60.72 ± 8.85	58.00 ± 7.92	56.59 ± 4.57	58.22 ± 6.77	53.89 ± 7.93	56.10 ± 10.01
BV (m^3^)	9.99 ± 4.34 ^a^	5.31 ± 2.00 ^c^	7.19 ± 2.87 ^b,c^	7.62 ± 2.12 ^a,b,c^	7.94 ± 2.97 ^a,b,c^	9.12 ± 4.55 ^a,b^
BV/TV (%)	16.38 ± 6.77 ^a^	9.18 ± 3.34 ^b^	12.55 ± 4.38 ^a,b^	13.21 ± 3.76 ^a,b^	14.40 ± 3.60 ^a^	15.99 ± 6.80 ^a^
Tb.Th (mm)	222.8 ± 50.5	230.5 ± 50.5	240.8 ± 61.4	224.8 ± 45.2	201.6 ± 40.4	218.1 ± 53.3
Tb.Sp (mm)	1.07 ± 0.18	1.22 ± 0.10	1.07 ± 0.22	1.13 ± 0.13	1.06 ± 0.18	1.06 ± 0.22
Tb.N (1/mm)	0.87 ± 0.26 ^a^	0.55 ± 0.17 ^b^	0.74 ± 0.20 ^a^	0.78 ± 0.18 ^a^	0.83 ± 0.18 ^a^	0.86 ± 0.26 ^a^
Ct.Th (mm)	0.56 ± 0.04 ^ab^	0.40 ± 0.05 ^d^	0.51 ± 0.05 ^c^	0.52 ± 0.0 ^c^	0.53 ± 0.03 ^b,c^	0.56 ± 0.06 ^a^
Ct.Ar (mm^2^)	9.06 ± 0.85 ^a^	6.03 ± 0.75 ^e^	7.77 ± 0.99 ^d^	8.00 ± 0.41 ^c,d^	8.26 ± 0.40 ^b,c,d^	8.74 ± 0.94 ^a,b^
Tt.Ar (mm^2^)	25.94 ± 2.93 ^a^	22.15 ± 2.27 ^b^	22.75 ± 2.90 ^b^	23.73 ± 1.38 ^b^	24.43 ± 2.09 ^a,b^	24.32 ± 3.32 ^a,b^
Ct.Ar/Tt.Ar (%)	35.64 ± 3.22 ^a^	28.15 ± 4.17 ^b^	35.21 ± 3.17 ^a^	34.42 ± 2.09 ^a^	34.44 ± 2.41 ^a^	36.81 ± 4.25 ^a^

Values are expressed as the Mean ± SD, *n* = 12/group. Different letters denote statistical differences among groups at *p*  <  0.05, as determined by ANOVA and LSD multiple-comparison test.

## Data Availability

All data generated during and/or analyzed are available from the corresponding author on reasonable request.

## Supplementary Materials

The following supporting information can be downloaded at: https://www.mdpi.com/article/10.3390/nu15153398/s1; Figure S1. Metabolic profile in calcium-deficient growing rats following MK-7 supplementation; Figure S2. Effects of MK-7 on the gut microbiota and metabolites in growing rats under calcium restriction; Table S1. Results of MK-7 on body weight and kidney coefficient in rats under calcium restriction; Table S2. Results of MK-7 on bone turnover markers of in rats under calcium restriction; Table S3. The overlapped three pathways due to calcium deficiency or MK-7 supplementation.
