# Supplementary material for: Effects of Menaquinone-7 on the Bone Health of Growing Rats under Calcium Restriction: New Insights from Microbiome-Metabolomics
Source: Nutrients. 2023 Jul 31;15(15):3398. doi: 10.3390/nu15153398 (PMC10421271; doi:10.3390/nu15153398)
Supplement: Supplementary file 1 [file nutrients-15-03398-s001.zip › nutrients-2461293-supplementary.pdf]

Effects of menaquinone-7 on the bone health of growing rats under calcium restriction: new insights from microbiome-metabolomics

Table S1. Results of MK-7 on body weight and Kidney coefficient in rats under calcium restriction

Group	Body weight (0 week) (g)	Body weight (13 week) (g)	Body weight gain (g)	Kidney weight(g)	Kidney coefficient(%bw)
NC	154.06±13.05	572.79±64.91 [a]	417.89±57.67 [a]	3.50±0.46	0.62±0.05 [b,c]
LC	152.20±13.31	493.51±40.31 [b]	341.31±40.45 [b]	3.55±0.43	0.70±0.05 [a]
RC	157.70±20.02	537.06±47.06 [a,b]	379.35±48.39 [a,b]	3.52±0.29	0.66±0.05 [a,b]
LMK	155.88±13.57	563.94±45.82 [a]	408.06±45.22 [a]	3.59±0.27	0.64±0.05 [b,c]
MMK	157.63±15.71	576.00±54.01 [a]	418.37±48.99 [a]	3.51±0.28	0.61±0.05 [c]
HMK	151.52±10.27	563.32±68.38 [a]	411.79±65.23 [a]	3.41±0.51	0.60±0.05 [c]

Values are expressed as the Mean ± SD. Different superscript letters in columns indicate significant differences among groups (p < 0.05) following ANOVA and LSD multiple-comparison test. n = 12/group. Rat groups consisted of low calcium administration (LC), normal calcium administration (NC), 75% normal calcium and 148% normal vitamin D_3 administration (RC), MK-7 treated (LMK, MMK, HMK, doses at 0.1, 1 and 10 mg/kg.bw, respectively) with feeding the RC diet.

Table S2. Results of MK-7 on bone turnover markers of in rats under calcium restriction

Group	PINP (ng/ml)	IGF-1 (pg/ml)
LC	4.41±1.98	159.29±52.47
NC	3.39±1.38	151.08±51.23
RC	3.49±0.89	160.68±55.04
LMK	3.14±0.95	151.52±42.39
MMK	3.17±0.59	145.49±49.03
HMK	3.87±1.29	136.50±36.52

Values are expressed as the Mean ± SD. Different superscript letters in columns indicate significant differences among groups (p < 0.05) following ANOVA and LSD multiple-comparison test. n = 12/group. Rat groups consisted of low calcium administration (LC), normal calcium administration (NC), 75% normal calcium and 148% normal vitamin D_3 administration (RC), MK-7 treated (LMK, MMK, HMK, doses at 0.1, 1 and 10 mg/kg.bw, respectively) with feeding the RC diet.

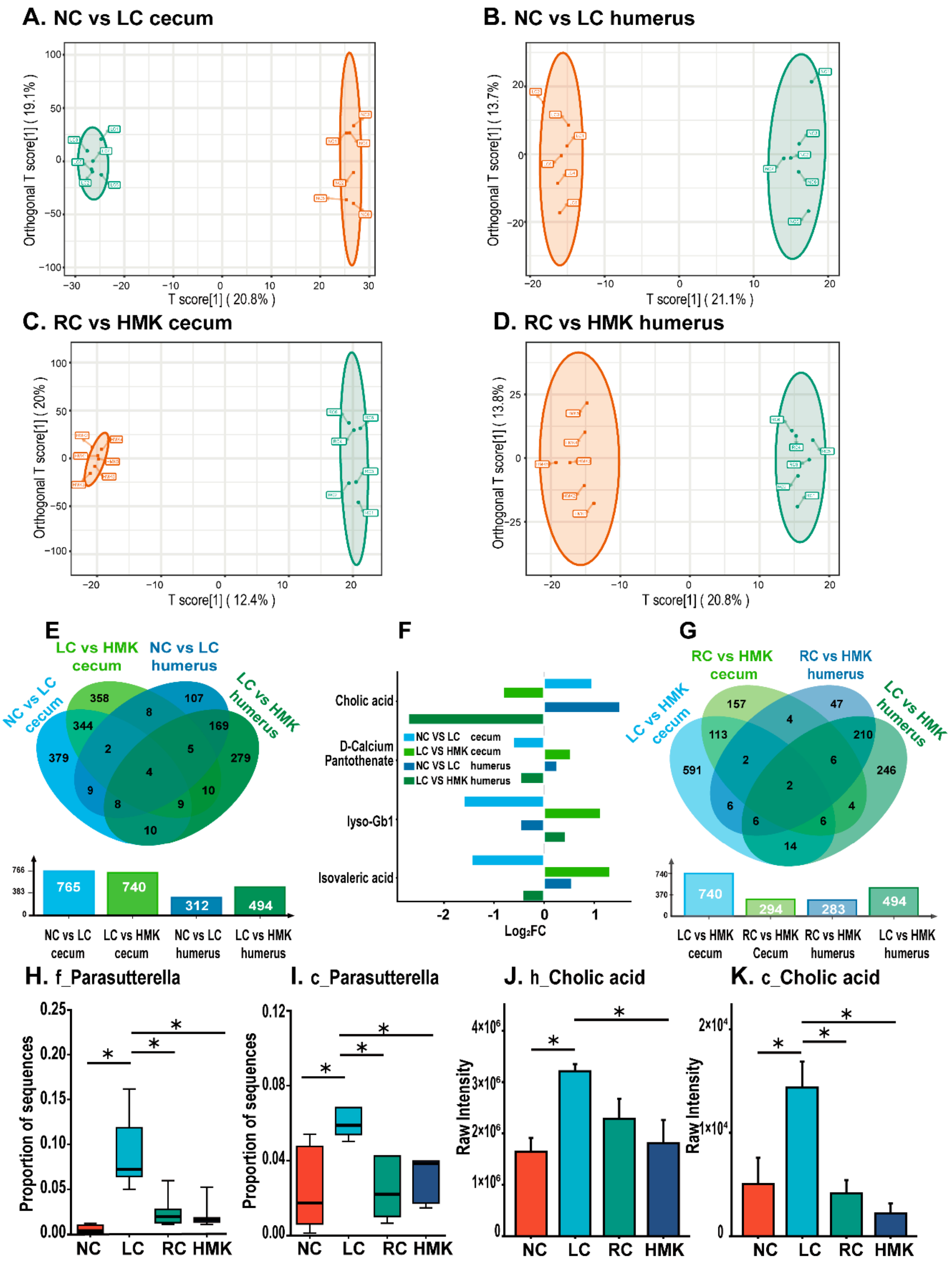

Figure S1. Metabolic profile in calcium-deficient growing rats following MK-7 supplementation (n = 6/group). **(A, B)** OPLS-DA score plots between the NC and LC groups' cecal content and humerus samples. **(C, D)** OPLS-DA score plots between the RC and HMK groups' cecal content and humerus samples. **(E)** A Venn diagram summarizing the significantly changed metabolites overlapping between the LC and either the NC or HMK group. **(F)** Barplots showing the four significantly overlapped altered metabolites (log2 fold change). **(G)** A Venn diagram summarizing the significantly changed metabolites overlapping between the HMK and either the LC or NC group. **(H,I)** The relative abundance of genues *Parasutterella* in the fecal and cecal content (t-test). **(J)** The raw intensity of cholic acid in the humerus (t-test). **(K)** The raw intensity of cholic acid in the cecal content sample (Wilcoxon test). The prefixes 'f', 'c', and 'h' before the flora and metabolites denote detection in feces, cecal content, and humerus, respectively.

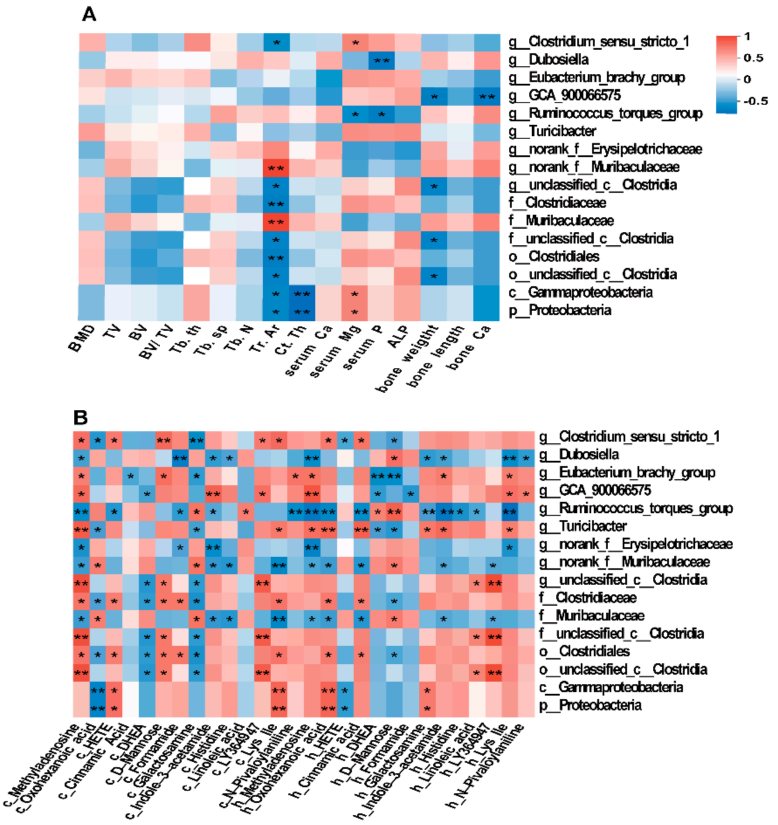

Figure S2. Effects of MK-7 on the gut microbiota and metabolites in growing rats under calcium restriction. **(A)** Correlation analysis of the bone quality and feces microbiota biomarker treated by MK-7. **(B)** Correlation analysis of the overlapped significant changes metabolites and fecal microbiota biomarker influenced by MK-7 treatment. Different color blocks represent Spearman correlation coefficients, where red indicates positive correlation, and blue indicates negative correlation. *$P < 0.05$; **$P < 0.01$. The prefixes 'f', 'c', and 'h' before the flora and metabolites denote detection in feces, cecal content, and humerus, respectively.

Table S3 The overlapped three pathways due to calcium deficiency or MK-7 supplementation

Pathway	Group	P value	Sample	Up metabolites[1]	Down metabolites
purine metabolism	NC vs LC	0.016	cecum	Urea, Adenine, 2'-Deoxyadenosine	Xanthine, L-Glutamine, Sulfate, Oxalic acid
		0.649	humerus	2'-Deoxyinosine, Uric acid, Cyclic GMP Xanthosine	-
	RC vs HMK	0.787	cecum	-	Urea
		0.003	humerus	Inosine, Guanosine, Hypoxanthine, Adenosine	Xanthosine, 2'-Deoxyinosine, Cyclic GMP,5-Hydroxyisourate, Guanine, Uric acid
protein digestion and absorption,	NC vs LC	0.490	cecum	Histamine	Tyrosine, L-Glutamine, L-Leucine, L-Lysine, Isovaleric acid, Tyramine
		0.042	humerus	Isovaleric acid, L-Tryptophan, L-Proline, Phenol, L-Phenylalanine, L-Isoleucine, L-Arginine, L-Histidine, L-Glutamic Acid	P-Cresol, Phenylalanine
	RC vs HMK	0.204	cecum	Isovaleric acid, Indole	L-Histidine, Histamine, Putrescine,
		0.000	humerus	-	L-Glutamic Acid, Phenol, L-Phenylalanine, L-Threonine, L-Serine, L-Isoleucine, L-Tyrosine, L-Arginine, DL-Leucine, L-Lysine, L-Tryptophan, L-Histidine, L-Proline, L-Methionine
aminoacyl-tRNA biosynthesis	NC vs LC	0.664	cecum	-	Tyrosine, L-Glutamine, L-Leucine L-Lysine
		0.050	humerus	L-Tryptophan, L-Proline, L-Phenylalanine, L-Isoleucine, L-Arginine, L-Histidine, L-Glutamic Acid	Phenylalanine
	RC vs HMK	0.897	cecum	-	L-Histidine
		0.000	humerus	-	L-Glutamic Acid, L-Phenylalanine, L-Threonine, L-Serine, L-Isoleucine, L-Tyrosine, L-Arginine DL-Leucine, L-Lysine, Cyclic GMP, L-Tryptophan, L-Histidine, L-Proline, L-Methionine

[1] The significantly increased (red) or decreased (green) metabolites were screened out according to VIP ≥ 1 and $p < 0.05$. “-” represents no signifant meabolite was detected.
